# Comparative analysis of glymphatic system alterations in multiple sclerosis and neuromyelitis optica spectrum disorder using MRI indices from diffusion tensor imaging

**DOI:** 10.1002/hbm.26680

**Published:** 2024-04-08

**Authors:** Minchul Kim, Inpyeong Hwang, Jung Hyun Park, Jin Wook Chung, Sung Min Kim, Ji‐hoon Kim, Kyu Sung Choi

**Affiliations:** ^1^ Department of Radiology Kangbuk Samsung Hospital, Sungkyunkwan University School of Medicine Seoul Republic of Korea; ^2^ Department of Radiology Seoul National University Hospital Seoul Republic of Korea; ^3^ Department of Radiology Seoul Metropolitan Government Seoul National University Boramae Medical Center Seoul South Korea; ^4^ Department of Neurology Seoul National University Hospital Seoul Republic of Korea

**Keywords:** diffusion tensor imaging, DTI‐ALPS, free water, glymphatics, multiple sclerosis, neuromyelitis optica spectrum disorder

## Abstract

**Objective:**

The glymphatic system is a glial‐based perivascular network that promotes brain metabolic waste clearance. Glymphatic system dysfunction has been observed in both multiple sclerosis (MS) and neuromyelitis optica spectrum disorder (NMOSD), indicating the role of neuroinflammation in the glymphatic system. However, little is known about how the two diseases differently affect the human glymphatic system. The present study aims to evaluate the diffusion MRI‐based measures of the glymphatic system by contrasting MS and NMOSD.

**Methods:**

This prospective study included 63 patients with NMOSD (*n* = 21) and MS (*n* = 42) who underwent DTI. The fractional volume of extracellular‐free water (FW) and an index of diffusion tensor imaging (DTI) along the perivascular space (DTI–ALPS) were used as indirect indicators of water diffusivity in the interstitial extracellular and perivenous spaces of white matter, respectively. Age and EDSS scores were adjusted.

**Results:**

Using Bayesian hypothesis testing, we show that the present data substantially favor the null model of no differences between MS and NMOSD for the diffusion MRI‐based measures of the glymphatic system. The inclusion Bayes factor (BF_10_) of model‐averaged probabilities of the group (MS, NMOSD) was 0.280 for FW and 0.236 for the ALPS index.

**Conclusion:**

Together, these findings suggest that glymphatic alteration associated with MS and NMOSD might be similar and common as an eventual result, albeit the disease etiologies differ.

**Practitioner Points:**

- Previous literature indicates important glymphatic system alteration in MS and NMOSD.
- We explore the difference between MS and NMOSD using diffusion MRI‐based measures of the glymphatic system.
- We show support for the null hypothesis of no difference between MS and NMOSD.
- This suggests that glymphatic alteration associated with MS and NMOSD might be similar and common etiology.

## Graphical abstract

We compared the glymphatic dysfunction of multiple sclerosis (MS) and neuromyelitis optica spectrum disorder (NMOSD) using MRI measures, specifically the fractional volume of extracellular‐free water and the DTI–ALPS index. We show support for the null hypothesis of no difference between glymphatic changes of MS and NMOSD. This suggests that glymphatic alterations in MS and NMOSD might be similarly influenced by a common etiology, yet it is important to consider the limitations in measurement methods and approach the results with caution.

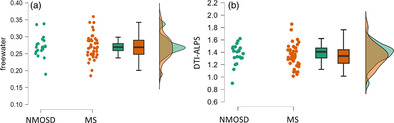

## 1 INTRODUCTION

**FIGURE 1:**
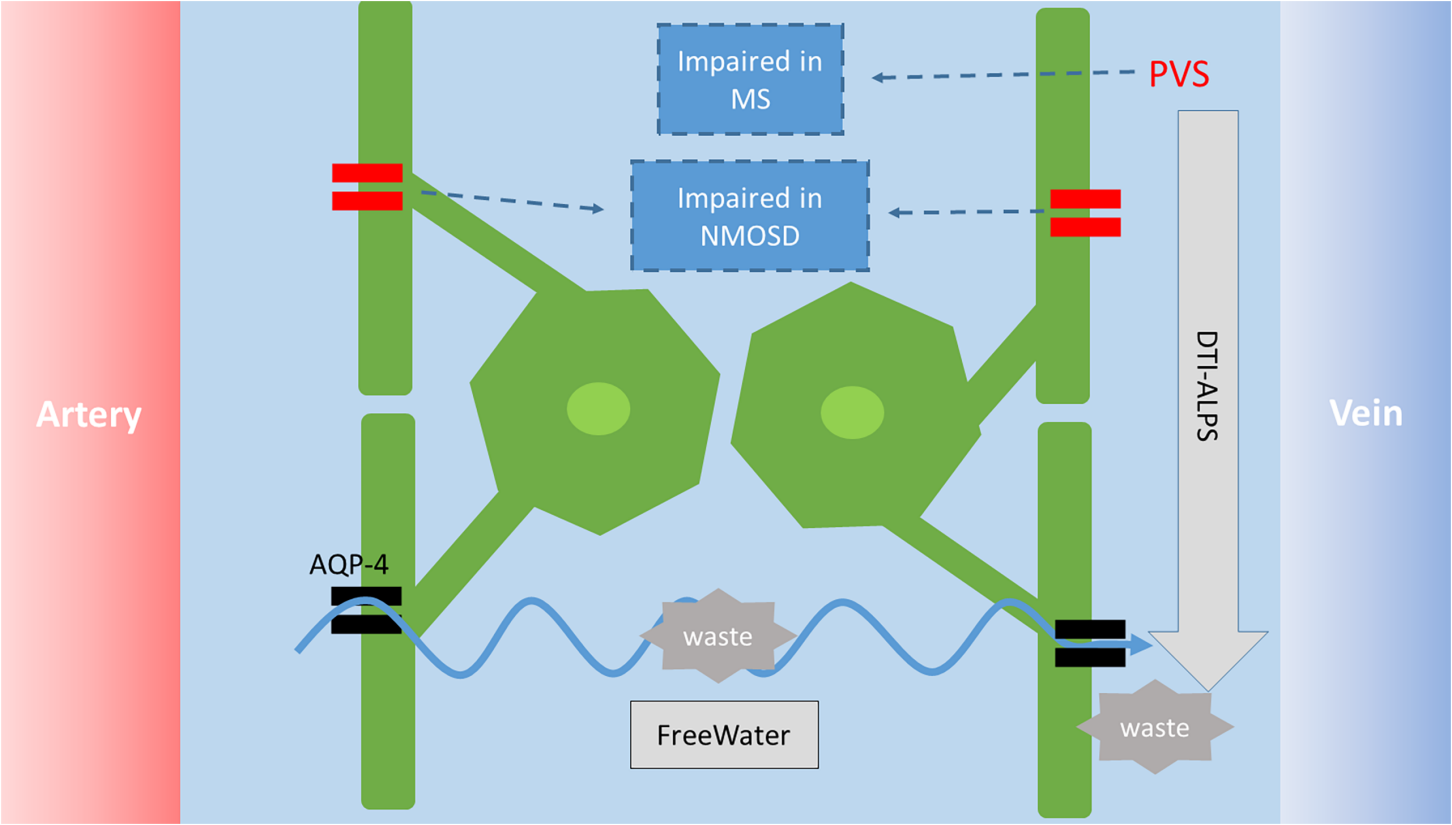
Schematic of glymphatic system and hypothetical dysfunction in multiple sclerosis (MS) and neuromyelitis optica spectrum disorder (NMOSD). Briefly, from arteries, CSF crosses the perivascular space entering the interstitium through the AQP‐4 channel and moving toward perivenous spaces (zigzag arrow). We hypothesize that perivenous inflammation in MS would alter perivascular flow, which is measured by DTI–ALPS index. While detorization of AQP‐4 is observed in NMOSD patients, which is the promoter of CSF flux through the glymphatic pathway. This detorization in the exchange of CSF and ISF would increase extracellular fractional volume of free water (FW) index. AQP‐4, aquaporin‐4; DTI–ALPS, diffusion along perivascular spaces; PVS, perivascular space.

The so‐called glia‐lymphatic or glymphatic system has been recently acknowledged as a brain waste clearance system. According to the glymphatic hypothesis (Xie et al., 2013), subarachnoid cerebrospinal fluid (CSF) enters the brain's interstitial space from the periarterial space through the aquaporin‐4 (AQP‐4) channel expressed in the astrocyte end‐feet. Then it mixes with the interstitial fluid (ISF) and waste solutes in the brain. The resulting CSF/ISF exchange and waste products are then drained out of the brain by the perivenous efflux pathway (Figure 1). Recently, promising MRI‐based noninvasive methods, namely, calculation of the fractional volume of free water (FW) in brain parenchyma (i.e., brain ISF) from a bitensor diffusion tensor imaging (DTI) model (Pasternak et al., 2009), and calculation of the diffusion along perivascular spaces (DTI–ALPS) index (Taoka et al., 2017) were introduced for the indirect evaluation of perivascular glymphatic activity (Andica et al., 2023; Kamagata et al., 2022).

Indeed, reduced the ALPS index was detected in several neurological disorders, including two major neuroinflammatory diseases of multiple sclerosis (MS) and neuromyelitis optica spectrum disorder (NMOSD) (Cacciaguerra et al., 2022; Carotenuto et al., 2022). Both of the disease entities showed significant relationship between disability and ALPS index, suggesting the direct correlation between glymphatic dysfunction and disease pathogenesis.

**FIGURE 2:**
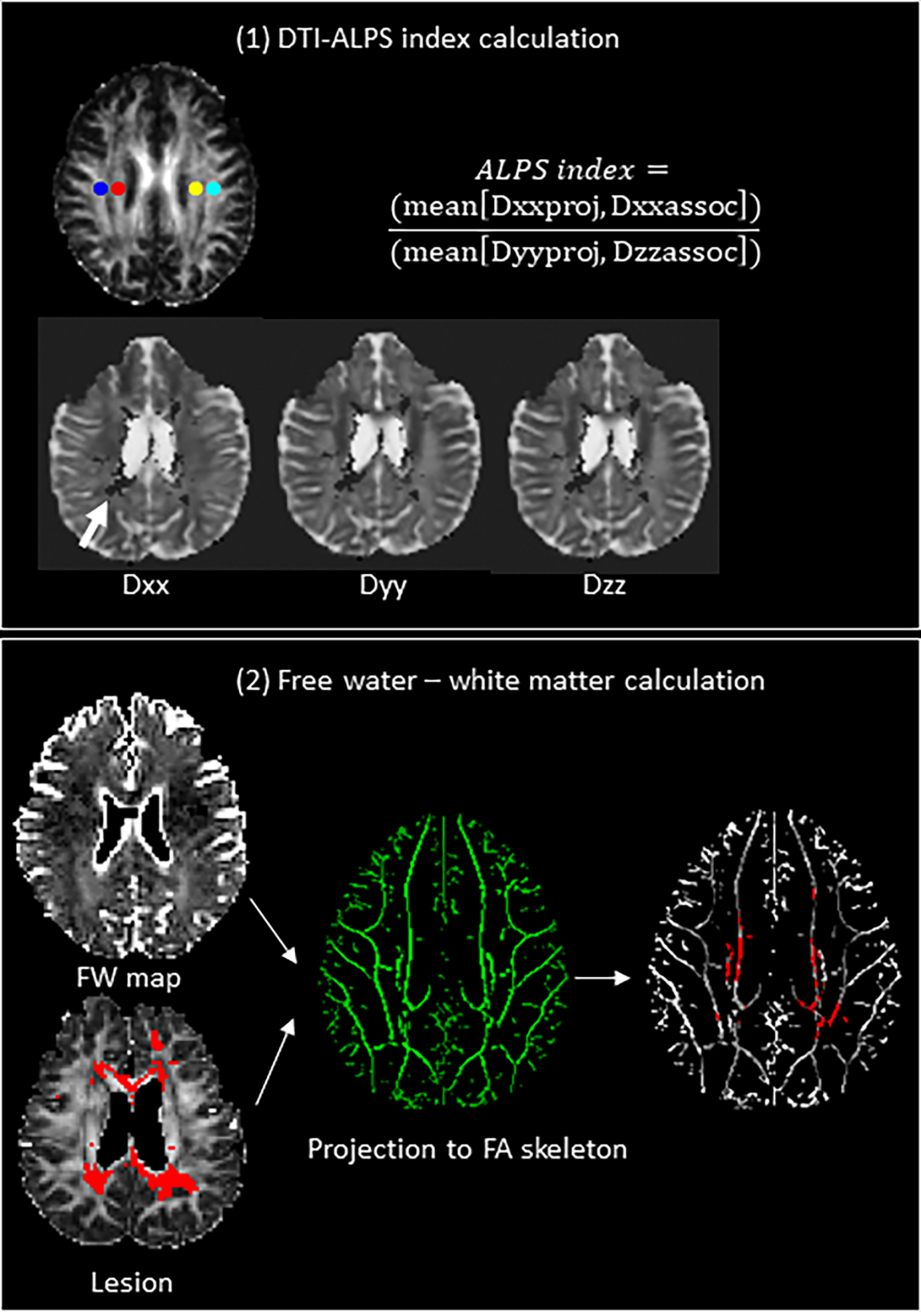
Schematic diagram of the study flow. Diffusion‐weighted images were obtained from all study participants and preprocessed. The preprocessed images were then used to calculate the (1) diffusion along perivascular spaces (DTI–ALPS) index and (2) white matter extracellular fractional volume of free water (FW). The ALPS index was calculated as follows: The FA map of each subject was co‐registered to the JHU‐ICBM‐FA template and the transformation matrix was applied to all the diffusivity maps and lesion maps. The pre‐definted ROIs for projection (superior corona radiata, blue and light blue) and association fibers (superior longitudinal fasciculus, red and yellow) at the level of lateral ventricle body were used to obtain the diffusivity values of $D_{{\mathrm{xx}}_{\text{proj}}}$, $D_{{\mathrm{xx}}_{\text{assoc}}}$, $D_{{\mathrm{yy}}_{\text{proj}}}$ and $D_{{\mathrm{zz}}_{\text{assoc}}}$. The lesion areas were excluded when obtaining *D*_*xx*_, *D*_*yy*_, *D*_*zz*_ values (white arrow). The FW was calculated as follows: a FW map was constructed based on a bi‐tensor model; a WM skeleton was constructed using a tract‐based spatial statistics pipeline; and the FW values and lesion maps were normalized to the WM skeleton. The FW values were averaged while lesions were used as the exclusion mask.

However, the theories supporting how the glymphatics function is altered should be different between MS and NMOSD, since they have different etiology. The pathophysiology and histopathology of MS is characterized by an inflammation crossing the blood–brain barrier via venous vessels leading to inflammation, demyelination, and neurodegeneration (Matthews et al., 2016). One histopathological hallmark of focal MS lesions is the perivascular (perivenous) inflammation pattern in the white matter (Fog, 1965). The ALPS index measures the decreased water diffusion along the perivascular space, by calculating the ratio of the diffusivity parallel to perivascular space around the deep medullary vein and the diffusivity in a perpendicular direction to major fiber tracts (Taoka et al., 2017) (Figure 2). Based on these facts, we hypothesized the pathophysiology of MS would alter the ALPS index more severely than NMOSD.

On the contrary, autoimmune antibody of AQP‐4 has been revealed to be a key pathology and diagnostic clue of NMOSD (Wingerchuk et al., 2015). AQP‐4 is required to maintain glymphatic function by promoting CSF movement from the perivascular spaces into the interstitial space and activating ISF flushing (Mestre et al., 2020). Elevated white matter FW has been suggested to reflect the stagnation of fluid drainage caused by glymphatic dysfunction (Andica et al., 2023; Kamagata et al., 2022). Therefore, AQP‐4 damage causes glymphatic impairment, including reduces in CSF‐ISF efflux (Andica et al., 2023). Based on these facts, we hypothesized the pathophysiology of NMOSD would increase the extracellular FW more severely than MS. In a nutshell, the current study aimed to investigate how the two neuroinflammatory diseases differently and specifically affect the human glymphatic system using FW and the ALPS index (Figure 1).

## 2 MATERIALS AND METHODS

### 2.1 Participants

Consecutive patients who visited the Seoul National University Hospital (SNUH) MS‐NMO clinic from April 2015 to April 2022 were prospectively enrolled in this study. This study was approved by the institutional review board of SNUH (IRB number: H‐1310‐083‐528), and informed consent was obtained from each participant who was willing to enroll in this study. All processes related to this study were conducted in accordance with the Declaration of Helsinki. The inclusion/exclusion criteria were as follows: (1) diagnosed with MS according to the McDonald criteria; (2) diagnosed with NMOSD with positive AQP4‐IgG according to the 2015 International Panel for NMO Diagnosis (IPND) criteria, with serum samples were tested for the presence of AQP4‐IgG using a live cell–based assay; (3) underwent MRI including DTI with three dimensional isotropic T2‐weighted FLAIR and three‐dimensional magnetization‐prepared rapid gradient‐echo (MPRAGE) T1‐weighted MRI, both of which allow thin‐section and high‐resolution imaging. The IPND criteria were as follows: at least 1 of the core clinical characteristics, with no other better explanation for their symptoms (or exclusion of alternative diagnoses), and the six core clinical characteristics include (1) optic neuritis; (2) acute myelitis; (3) area postrema syndrome (i.e., nausea, vomiting, hiccups); (4) acute brainstem syndrome; (5) symptomatic narcolepsy or acute diencephalic syndrome with typical MRI lesion(s); and (6) symptomatic cerebral syndrome with typical MRI lesion(s) (Wingerchuk et al., 2015). Among NMOSD patients, 7 had previous optic neuritis, 18 had previous myelitis, and 2 with history of brain syndrome. Patients were evaluated during the remission phase of the disease (i.e., at least 8 weeks apart from clinical relapse and intravenous steroids administration and have a stable treatment or no treatment for at least 6 months (Cacciaguerra et al., 2022; Carotenuto et al., 2022). Finally, 63 patients were enrolled in the present study (*n* = 42 in the MS group and *n* = 21 in the NMOSD group). Clinical characteristics, including age, sex, expanded disability status scale (EDSS) scores, treatment history prior to MRI, and disease duration, were collected from the electronic medical record system of the hospital (Table 1).

**TABLE 1:** Clinical characteristics of study population.

	NMOSD (*n* = 21)	MS (*n* = 42)	*p*‐value
Age (years)	47.23 ± 11.18	33.97 ± 7.52	.001*
Gender (Male: Female)	2:19	17:25	.012*
Disease duration (years)	3.64 ± 3.93	5.01 ± 5.24	.294
EDSS	2.95 ± 2.79	1.48 ± 1.65	.011*
Number of relapses (mode, range)	2 [0,8]	2 [0,8]	
Lesion volume in MNI space (mL, natural logarithm)	6.25 ± 2.36	8.26 ± 1.38	.04*
Gray matter volume (mL)	585.4 ± 88.9	568.9 ± 101.1	.29
White matter volume (mL)	398.7 ± 103.3	413.8 ± 82.5	.18
Treatment			
Immunosoppressants (%)	17 (80.9%)	28 (66.7%)	
Oral steroids (%)	21 (100%)	33 (78.5%)	
Rituximab (%)	14 (66.7%)	—	
Disease modifying therapy (%)	—	37 (88.1%)	

Abbreviation: EDSS, Expanded Disability Status Scale.

* *p* < .05 indicates statistical significance.

### 2.2 MRI data acquisition and segmentation

All MR images were acquired using 3.0 T MR scanner (Ingenia CX, Philips Healthcare, Best, the Netherlands) with a conventional head gradient coil. T2 FLAIR imaging, T1‐weighted imaging, and DTI were acquired with the following scan parameters: (1) three‐dimensional (3D) isotropic fast‐ spin echo sagittal FLAIR T2‐weighted sequence (repetition time [TR] = 4800 ms, echo time [TE] = 265 ms, inversion time = 1650 ms, echo train length = 175, field of view [FOV] = 230 mm, matrix = 230 × 230, and voxel size = 1 × 1 × 1 mm); (2) 3D high‐resolution T1‐weighted sequence (TR = 9.8 ms, TE = 4.5 ms, inversion time = 1650 ms, flip angle = 8°, FOV = 230 mm, matrix = 230 × 230, slice thickness = 0.5 mm, no gap, and voxel size = 1 × 1 × 0.5 mm); and (3) spin‐echo single‐shot echo‐planar imaging DWI sequence (TR = 9500 ms, TE = 75 ms, number of excitations = 1, matrix = 128 × 128, FOV = 230 × 230 mm, number of slices = 80, slice thickness = 2 mm, slice gap = 0 mm, orientation = axial, *b* = 1000 s/mm^2^ and one additional b0‐volume). We used 32 nonlinear diffusion weighting gradient directions to estimate the intensity and direction of the diffusion anisotropy. The white matter lesion regions of interest (ROI) were manually drawn section by section on the FLAIR sequence by 2 authors (K.J.H. and I.H.). We quantified tissue volumes of the brain using FAST software in with FMRIB's software library (FSL) (Jenkinson et al., 2012; Zhang et al., 2001).

### 2.3 Freewater calculation

The DTI images underwent artifact corrections using Marchenko‐Pastur Principal Component (MP‐PCA) denoising algorithm and Gibbs unringing using MRtrix3 command line “dwidenoise” and “mrdegibbs,” corrections of eddy currents and movements were accomplished with FSL command line “eddy” (Tournier et al., 2019). We visually checked the quality of DWI and the automated quality‐assessment protocols for DWI, that is, the temporal signal‐to‐noise ratio (tSNR), was assessed for each of the participants. Regarding tSNR, a method used to quickly screen the overall data quality, the participants’ lowest value was 7.57, which is above the suggested cutoff value (6.47) for poor data (Roalf et al., 2016). Free‐water corrected DTI maps were calculated using an in‐house MATLAB script that was previously used in this study cohort (Bergamino et al., 2017; Bergamino et al., 2021; Kim et al., 2022). Finally, we acquired the mean of the FW index of each participants’ white matter skeleton (Andica et al., 2023) (Figure 2).

### 2.4 ALPS index calculation

We used an automated method to calculate the DTI–ALPS index (Liu et al., 2023). Using DWI images, the FA map and *x*‐, *y*‐ and *z*‐axis diffusivity maps were generated using FSL command line “dtifit.” The FA map of each subject was co‐registered to the JHU‐ICBMFA template and the transformation matrix was applied to all the diffusivity maps by using FSL command line “flirt.” The projection and association fibers at the level of lateral ventricle body were recognized as the superior corona radiata (SCR) and the superior longitudinal fasciculus (SLF) based on the JHU‐ICBM‐DTI‐81‐white matter Labeled Atlas and the ROIs were automatically defined as spheres with 5 mm diameter in the areas of bilateral projection fibers (superior corona radiata, Figure 2, blue and light blue) and association fibers (superior longitudinal fasciculus, Figure 2, red and yellow) which applied on all subjects’ diffusivity maps. The diffusivity values of *D*_*xx*_, *D*_*yy*_ and *D*_*zz*_ of bilateral SLF and SCR were automatically outputted for the ALPS index calculation (Liu et al., 2023). The lesion areas were excluded when obtaining *D*_*xx*_, *D*_*yy*_, *D*_*zz*_ values (Figure 2). The ALPS index is defined by the average of bilateral ALPS indexes (mean ALPS index), which is by the ratio of the mean of *x*‐axis diffusivity in the area of projection fibers ($D_{{\mathrm{xx}}_{\text{proj}}}$) and *x*‐axis diffusivity in the area of association fibers ($D_{{\mathrm{xx}}_{\text{assoc}}}$) to the mean of the *y*‐axis diffusivity in the area of projection fibers ($D_{{\mathrm{yy}}_{\text{proj}}}$) and *z*‐axis diffusivity in the area of association fibers ($D_{{\mathrm{zz}}_{\text{assoc}}}$) as follows (Taoka et al., 2017):

$\text{ALPS index}=\frac{\text{mean}\left(D_{{\mathrm{xx}}_{\text{proj}}},D_{{\mathrm{xx}}_{\text{assoc}}}\right)}{\text{mean}\left(D_{{\mathrm{yy}}_{\text{proj}}},D_{{\mathrm{zz}}_{\text{assoc}}}\right)}$

### 2.5 Statistical analyses

Statistical analyses were performed in JASP team (2023). Two separate Bayesian ANCOVAs with FW, ALPS index values as a dependent variable, age and EDSS scores as covariates, and group (MS, NMOSD) as a fixed factor were conducted. Age and EDSS were chose as there are previous studies reporting its possible association to glymphatic alteration (Carotenuto et al., 2022; Gullett et al., 2020). JASP give the report of Bayesian model comparisons, to allow quantifying the degree of evidence for a given model compared to the null and other models. In addition, we computed the model‐averaged inclusion probabilities of each predictors, and calculated the inclusion Bayes factor. The Bayes factor (BF_01_) is reported to quantify the evidence for H1 (alternative hypothesis) relative to H0 (null hypothesis) (Wagenmakers et al., 2017). A BF_01_ of above 3 indicates substantial evidence for H_1_, whereas a BF_01_ of below 1/3 indicates substantial evidence for H0, and between these values indicates that the data are insensitive (Dienes, 2014). The inclusion Bayes factor quantifies the change from prior inclusion odds to posterior inclusion odds and can be interpreted as the evidence in the data for including a predictor (van den Bergh et al., 2020).

## 3 RESULTS

### 3.1 Free water index

**FIGURE 3:**
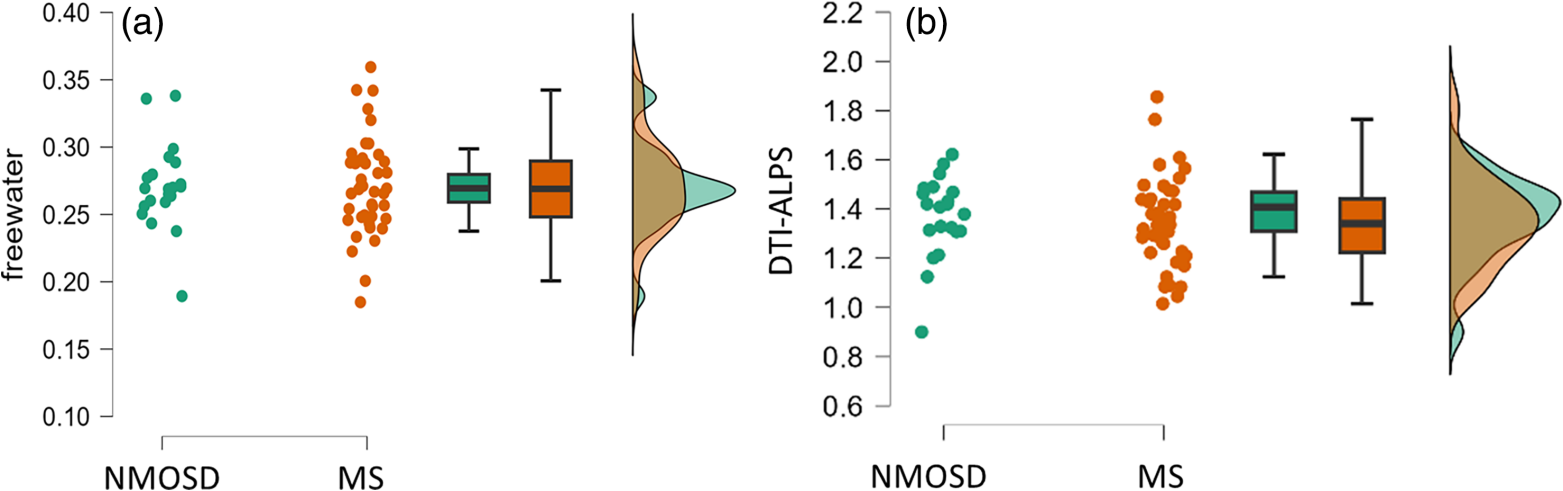
Between‐group differences in diffusion magnetic resonance imaging measurements. Shown are rain cloud and box plots of (a) the free water and (b) diffusion along the perivascular space (DTI–ALPS) index between the neuromyelitis optica spectrum disorder (NMOSD) and multiple sclerosis (MS). The distributions are similar for both measures.

Bayesian ANCOVA with FW as a dependent variable, age and EDSS score as covariates, and group (MS, NMOSD) as a fixed factor showed moderate evidence for the null model (i.e., no difference between groups) over the alternative (BF_M_ = 5.99, *p* (M|Data) = .46; Figure 3a). The prior odd for the null model was 0.125. The inclusion Bayes factor (BF_10_) of model‐averaged probabilities of group (MS, NMOSD) was 0.280, which means the data are about 3.43 times more likely under the models that exclude the group predictor than under the models with this predictor.

### 3.2 DTI–ALPS index

Bayesian ANCOVA with DTI–ALPS index as a dependent variable, age and EDSS score as covariates, and group (MS, NMOSD) as a fixed factor again showed moderate evidence for the null model (i.e., no difference between groups) over the alternative (BF_M_ = 6.32, *p* (M|Data) = .47; Figure 3b). The inclusion Bayes factor (BF_10_) of model‐averaged probabilities of group (MS, NMOSD) was 0.236, which means the data are about 4.36 times more likely under the models that exclude the group predictor than under the models with this predictor.

## 4 DISCUSSION

In this study, we aimed to differently understand the glymphatic alteration in the two prominent neuroinflammatory diseases of MS and NMOSD. Based on the disease etiology, we hypothesized that different aspects of glymphatic system would be affected. As MS is characteristic of perivascular inflammation, ALPS index would be more decreased. On the contrary, while NMOSD affects AQP‐4 which would more increase FW index. We used Bayesian hypothesis testing to quantify evidence for the null or alternative hypothesis. Against our hypothesis, the result supported the null hypothesis of no difference in ALPS index nor FW index between the MS and NMOSD.

The absence of a difference in indirect glymphatic measures between MS and NMOSD patients may cautiously be interpreted as support for the hypothesis of no underlying difference in glymphatic alteration between these groups. Although the etiologies differ, the consequence of long standing glymphatic deterioration may eventually reach similar values. In a previous study reporting ALPS index decrease in MS, ALPS index decreased up to about 4 years from disease onset, but after this breakpoint the value was plateaued (Carotenuto et al., 2022). Another interpretation we provide is that glymphatic system alteration is an interconnected process that is not undergo separately. To elaborate, extracellular FW accumulation does not only increase FW itself but the flood of water affect glymphatic flow either, thus decrease ALPS index. Oppositely, altered glymphatic flow in the perivascular space not only decreases ALPS index, but also result in extracellular fluid accumulation thus increase FW index. In our point of view, our study is firstly reporting the possibility of no difference in indirect indicators of the glymphatic system, and suggesting these measures are connected, rather than separately affected. In the other words, although the etiologies differ, the consequences of neuroinflammatory processes may partly shared between MS and NMOSD.

But, there can be critics of our study, because the indirect indicators of the glymphatic system are not specific to the glymphatic alteration nor reflects whole brain glymphatic alteration status. Notably, the ALPS index does not exclusively measure the diffusivity of the perivenous space around the deep medullary vein – that is, it is also influenced by the surrounding white matter microstructure included in the ROI. Although we excluded the white matter lesions from analysis, axonal loss and glial cell activity changes are suggested in the normal‐appearing white matter in MS (Dekker & Wattjes, 2017). Similarly, the changes in FW index can occur through different physiologic mechanisms, such as atrophy, edema, Alzheimer's Disease, a reduction in myelin content, or modulation in the permeability of the blood–brain barrier (Kamagata et al., 2022).

In summary, we report that no difference of FW and ALPS indices between individuals with MS and NMOSD, which might reflect similar degrees of glymphatic system impairment. Our findings provide evidence of shared consequence of glymphatic impariment, albeit the disease etiologies differ. However, considering the methodological limitations of this study, with regard to low specificity in particular, the results should be interpreted with caution.

## AUTHOR CONTRIBUTIONS

Study design, MCK, KSC, JK, and SMK; Data collection, JHP, JWC, IH, JK, and SMK; Data analysis and interpretation, MCK, KSC, JK, and SMK; Figures, MCK, KSC; Manuscript writing, MCK and KSC. All authors revised and approved the final version of the manuscript.

## FUNDING INFORMATION

This work was supported by the National Research Foundation of Korea (NRF) grant funded by the Korea government (MSIT) (No. RS‐2023‐00251022) (K.S.C); the Phase III (Postdoctoral fellowship) grant of the SPST (SNU‐SNUH Physician Scientist Training) Program (K.S.C); and the SNUH Research Fund (No. 04‐2023‐2050) (K.S.C.); the Technology Innovative Program (20011878), Development of Diagnosis Medical Devices with Artificial Intelligence Based Image Analysis Technology) funded by the Ministry of Trade, Industry & Energy (MOTIE, Korea) (J.W.C)

## CONFLICT OF INTEREST STATEMENT

The authors disclose no conflicts of interest related to this work.

## Data Availability

The data that support the findings of this study are available on request from the corresponding author. The data are not publicly available due to privacy or ethical restrictions.
